# Repetitive Transcranial Magnetic Stimulation Improved Source Memory and Modulated Recollection-Based Retrieval in Healthy Older Adults

**DOI:** 10.3389/fpsyg.2020.01137

**Published:** 2020-06-19

**Authors:** Xiaoyu Cui, Weicong Ren, Zhiwei Zheng, Juan Li

**Affiliations:** ^1^CAS Key Laboratory of Mental Health, Center on Aging Psychology, Institute of Psychology, Chinese Academy of Sciences, Beijing, China; ^2^Department of Psychology, University of Chinese Academy of Sciences, Beijing, China; ^3^Department of Psychology, Hebei Normal University, Shijiazhuang, China; ^4^Magnetic Resonance Imaging Research Center, Institute of Psychology, Chinese Academy of Sciences, Beijing, China

**Keywords:** older adults, source memory, repetitive transcranial magnetic stimulation, recollection, event-related potentials

## Abstract

Source memory is one of the cognitive abilities that are most vulnerable to aging. Luckily, the brain plasticity could be modulated to counteract the decline. The repetitive transcranial magnetic stimulation (rTMS), a relatively non-invasive neuro-modulatory technique, could directly modulate neural excitability in the targeted cortical areas. Here, we are interested in whether the application of rTMS could enhance the source memory performance in healthy older adults. In addition, event-related potentials (ERPs) were employed to explore the specific retrieval process that rTMS could affect. Subjects were randomly assigned to either the rTMS group or the sham group. The rTMS group received 10 sessions (20 min per session) of 10 Hz rTMS applying on the right dorsolateral prefrontal cortex (i.e., F4 site), and the sham group received 10 sessions of sham stimulation. Both groups performed source memory tests before and after the intervention while the electroencephalogram (EEG) was recorded during the retrieval process. Behavioral results showed that the source memory performance was significantly improved after rTMS compared with the sham stimulation; ERPs results showed that during the retrieval phase, the left parietal old/new effect, which reflected the process of recollection common to both young and old adults, increased in the rTMS group compared with the sham stimulation group, whereas the late reversed old/new effect specific to the source retrieval of older adults showed similar attenuation after intervention in both groups. The present results suggested that rTMS could be an effective intervention to improve source memory performance in healthy older adults and that it selectively facilitated the youth-like recollection process during retrieval. This study was registered in the Chinese Clinical Trial Registry (ChiCTR) with the identifier chictr-ire-15006371.

## Introduction

Fluid cognitive abilities (e.g., executive function, processing speed, sensory function, long-term memory, and reasoning) have been shown to decline with aging in numerous studies (for reviews, see Hofer and Alwin, 2008; Craik and Salthouse, 2011). One of the most common subjective complaints among the elderly is a decline in episodic memory. Previous studies have demonstrated that different forms of episodic memory might be impaired differently during aging (Old and Naveh-Benjamin, 2008). Compared with single items or content, age-related memory decline is frequently presented as fail to remember associative or contextual information. The elderly exhibit relative inability to form and retrieve links among two representations (e.g., two items, item and related context, and two contextual codes). Naveh-Benjamin (2000) put forward the associative deficit hypothesis (ADH), which suggested that the impairment of episodic memory with aging was mostly due to the impairment of memorizing associative information instead of item information. Dual-process theory provided a potential explanation for the age-related associative memory deficits. Specifically, the dual-process theory suggests that there are two distinct processes during episodic retrieval, which are named familiarity and recollection (Yonelinas, 2002; Rugg and Curran, 2007). Familiarity is relatively fast, with little or no contextual details retrieved; recollection is rather slow and involves the retrieval of some specific item-related contextual information (see Rugg and Yonelinas, 2003 for reviews). Both processes can support item retrieval, whereas the retrieval of contextual information or associative information mainly relays on recollection. Therefore, the inability to retrieve contextual information or associative information could be due to the impaired recollection process with aging (Daselaar et al., 2006; Friedman, 2013; Koen and Yonelinas, 2014).

Given the fact that the episodic memory declines with aging, numerous researchers have focused on how to mitigate and remediate age-related damage. Over the past decades, plenty of studies have demonstrated that the memory abilities of healthy older adults can be enhanced through cognitive or physical training (Park and Bischof, 2013; Gutchess, 2014; Ballesteros et al., 2015). In particular, mnemonic strategy training (Kirchhoff et al., 2012; Dresler et al., 2017) and aerobic exercise (Erickson et al., 2011) could effectively facilitate the memory performance of older adults. Recently, non-invasive brain stimulation techniques have been applied to aged adults to enhance declining functions and learning abilities (Zimerman and Hummel, 2010; Vallence and Goldsworthy, 2014). One of non-invasive brain stimulation technique is repetitive transcranial magnetic stimulation (rTMS), which could repetitively produce brief magnetic pulses that induce an electric field in the brain and further depolarize neurons (Rossi et al., 2009). When TMS is applied with the appropriate parameter (e.g., pulse frequency, duration, and amplitude), the currents could have a neuro-modulatory effect during (online) and beyond (off-line) the stimulation period (Wagner et al., 2009). whereas the online stimulation (stimulated during a task) usually helps to address causal relations between the role of specific brain regions and specific behavior processes. The off-line paradigm (Robertson et al., 2003), which means magnetic pulses are administered during a rest period, could be used as an interventional strategy. Depending on the stimulation parameters, rTMS can enhance or suppress cortical excitability in the targeted area of cortex (Rossi and Rossini, 2004; Hsu et al., 2015). Generally, cortical excitability would be enhanced by high-frequency rTMS (≥5 Hz) and suppressed by low-frequency rTMS (≤1 Hz) (Fregni and Pascual-Leone, 2007).

Off-line rTMS is most commonly used as a treatment for neuropsychiatric diseases, such as depression (Martin et al., 2017), schizophrenia (Hasan et al., 2016), and addiction (Qiao et al., 2016). For older adults, rTMS has been commonly applied to improve memory, specifically the episodic memory, in Alzheimer’s disease patients and mild cognitive impairment (MCI) patients (Cotelli et al., 2012; Turriziani et al., 2012; Drumond Marra et al., 2015; Koch et al., 2018). Most of these studies showed consistently an improvement in episodic memory performance after off-line rTMS. Considering about the target regions, although the medial temporal lobe (MTL) has been shown to play an essential role in episodic memory, owing to the limited spatial ability to target the deep region, most rTMS studies chose the targeting neocortical regions, such as the parietal cortex, which is functionally connected with the MTL (Wang et al., 2014). In most rTMS protocols in the elderly, the dorsolateral prefrontal cortex (DLPFC) is the main stimulation site; one obvious reason is that DLPFC is the most accessible cortex for the TMS coil (Slotema et al., 2010; Iimori et al., 2019). Besides, older adults tend to over-recruit the prefrontal cortex (PFC), and the pattern of bilateral PFC recruit has been observed in different cognitive tests, including episodic memory (Cabeza et al., 2002; Rosen et al., 2002; Davis et al., 2012). Previous studies showed that when rTMS was applied to disrupt the DLPFC, the episodic memory would be impaired in older adults (Rossi et al., 2004; Manenti et al., 2011). Therefore, for the studies interested in episodic memory in the elderly, DLPFC also became the first-choice stimulation site.

Only a few studies explored the rTMS effect on episodic memory in healthy older adults and found inconsistent results for the rTMS effect. For example, Solé-Padullés et al. (2006) applied 5 min off-line high-frequency rTMS on DLPFC in the elderly, and the results found that rTMS significantly improved the associative memory. Peña-Gomez et al. (2012) applied 5 Hz high-frequency off-line rTMS for 5 min over the PFC in healthy elderly adults; the results demonstrated that rTMS could improve associated memory performance in normal aging individuals, regardless that whether the individuals were APOE carriers or not. However, Vidal-Piñeiro et al. (2014) used off-line TMS targeting the left inferior frontal gyrus, and the results showed no difference in recognition memory accuracy between the TMS group and the sham group. Davis et al. (2017) conducted both low-frequency (1 Hz) and high-frequency (5 Hz) rTMS in healthy older adults, the stimulation site was the left DLPFC, and the results showed no differences in recognition memory performance between 1 and 5 Hz rTMS. The current contradictory results make it difficult to conclude that rTMS could improve the cognitive ability in healthy elderly, and more studies are needed. The reason for the contradictory results might be that some studies have used single-session stimulation while others have used multi-session stimulation. Yeh and Rose (2019) reviewed all the rTMS studies on episodic memory, and they proposed that the implementation of high-frequency multi-session off-line rTMS could promote episodic memory in MCI and AD patients. Only a few studies have implemented multi-session off-line stimulation in healthy older adults. Therefore, it is also important to inspect the multi-session off-line rTMS effects with healthy elderly. Another limitation of the existing studies is that it is difficult to know which specific memory process rTMS could modulate. Many studies in the elderly used the standard neuropsychology test, which is hardly to distinguish the different processes of memory. Even for studies that used classical memory paradigm, no method was used to detect which process (especially the distinct process during retrieval) was promoted by rTMS.

Event-related potentials (ERPs) have been widely used in investigating the memory-related processes. Some particular ERP components were found to correlate with distinct retrieval processes in young adults. For example, the early old/new effect occurs approximately 300–500 ms after the onset of the stimulus and typically maximal over frontal sites, which is considered to index familiarity (Curran, 2000; Curran and Hancock, 2007, but see Paller et al., 2007). The late old/new effect observed around the 500–800 ms poststimulus onset and most pronounced at the left parietal sites, also called LPC, is hypothesized to reflect recollection (Rugg and Curran, 2007; Bridger and Mecklinger, 2012). Besides, there is another ERP component frequently observed in episodic memory studies and considered as an index of post-retrieval processes – the late posterior negativity (LPN). LPN mostly occurred later than LPC and takes the form of a prominent reversed old/new effect at posterior recording sites. The previous study suggested that the LPN reflects a reconstructive process, which often appeared when the specified contextual information cannot easily be recovered (for a review, see Johansson and Mecklinger, 2003; Mecklinger et al., 2016). In older adults, previous studies demonstrated that the ERP components related to retrieval were somewhat different from what was observed in young participants. For the ERP component correlation to familiarity, some researchers found this early frontal maximal old/new effect presented with right-lateralized topography in older adults (Wegesin et al., 2002; Ally et al., 2008; Scheuplein et al., 2014, but see Wang et al., 2012). For the ERP component correlated with recollection, although some studies identified the similar 500–800 ms left parietal old/new effects in older as in young adults (Mark and Rugg, 1998; Duarte et al., 2006; Zheng et al., 2016), other studies failed to find the recollection-related left parietal old/new effect in older adults; instead, they found an onset earlier and more widespread late negative component (Li et al., 2004; Wiese et al., 2012; Dulas and Duarte, 2013; Wolk et al., 2013; Kamp and Zimmer, 2015; Horne et al., 2020). The functional significance of the late negative component in older adults was considered to be different from that of younger adults. Instead of reflecting a reconstructive process, the late negative component might also correlate with the recollection-based retrieval process in older adults (Li et al., 2004; Horne et al., 2020).

In the present study, the first aim is to investigate whether the use of high-frequency rTMS intervention in healthy elderly can improve episodic memory ability. The second aim is to detect which specific retrieval process would be influenced after the intervention. We randomly assigned half of the subjects into the rTMS group, in which subjects received 10 sessions of 10 Hz rTMS targeting the right DLPFC. The other half became the control group and received 10 sessions of sham stimulation. Both groups were performed a source memory test before and after the stimulation while electroencephalogram (EEG) was recorded during the retrieval process. We hypothesize that source memory would be improved after the rTMS intervention in healthy elderly. Correspondingly, we assume that the recollection process of the elderly will be modulated by rTMS. But it is open as to which recollection-based ERP components (the left parietal old/new effect and/or the late reversed old/new effect) will change after intervention.

## Materials and Methods

### Participants

**FIGURE 1:**
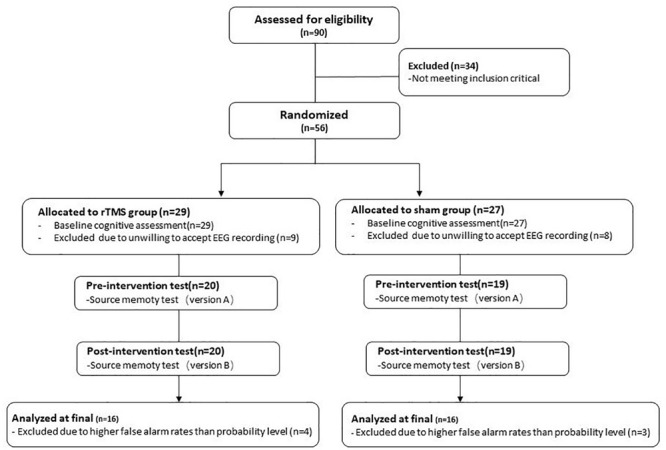
The flowchart of the repetitive transcranial magnetic stimulation (rTMS) intervention.

Ninety healthy older participants speaking Chinese as their native language registered with the present study from the First Hospital of Heibei Medical University. The inclusion criteria of participants were following: (1) aged 60 or older, (2) at least 8 years of education, (3) global cognitive function at normal level [the score of Beijing version of the Montreal Cognitive Assessment (MoCA-BJ) ≥ 21; Yu et al., 2012], (4) no subjective memory complaints (score ≥ 2 on Memory Complaint Assessment; Lam et al., 2005), (5) no depressive symptoms [the score of Center for Epidemiologic Studies Depression Scale (CES-D) ≤ 16; Roberts and Vernon, 1983], (6) right-dominant hand, and (7) passed safety screening of MRI/TMS (Rossi et al., 2009) and met the safety standard of MRI scan and TMS intervention. Participants with a history of neurological or psychiatric disorders or traumatic brain injury were excluded. After baseline evaluation, 34 participants were excluded from the study because they did not meet the inclusion criteria. Of the remaining 56 participants, 39 participants were willing to accept EEG data recording, with 20 in the rTMS group and 19 in the sham group. Thirty-nine participants all finished corresponding intervention plans. Consequently, the present study only reports the results of these 39 participants. During the process of data analysis, subjects who have higher false alarm rates than probability level were excluded. Finally, 32 participants were included in the final analysis, with 16 in the rTMS group and 16 in the sham group (see Figure 1).

All participants signed informed consent documents before participating in the present study and were paid upon completion of the study. The Ethics Committee of the Institution of Psychology, Chinese Academy of Sciences, approved the present study. Meanwhile, the study was registered in the Chinese Clinical Trial Registry (ChiCTR) with the identifier chictr-ire-15006371.

### Procedure

The present study conducted a randomized clinical trial with a single-blind and sham-controlled procedure. Participants were randomly assigned to either the rTMS group or the sham group. Participants were blind to the study design and grouping arrangement. Before the recruitment, we generated 60 random numbers with an expectation of recruiting 60 participants. Each participant was randomly assigned to a number. Participants with odd numbers formed as rTMS group, and participants with even numbers formed as sham group.

Participants of rTMS group received 10 sessions (20 min/session) of rTMS intervention in 2 weeks. Participants of the sham group received the same intervention plan, but the coil was placed vertically onto their scalp (Kim et al., 2012). This way of placement can ensure that magnetic fields scarcely or never pass through the brain but can generate the same sound effect and vibration effect as compared with those in the rTMS group. Therefore, the subjects in the two groups had the same feelings, and the subjects could not guess their own grouping from the intervention process.

At baseline, all participants completed demographic questionnaires and Cognitive Function Batteries including Verbal Ability, Comprehensive Executive Function, and Working Memory Span. Those test data were not analyzed and reported in the present study because they were not current concerns. Additionally, participants completed a source memory test (Version A) at baseline. Then, the rTMS group and sham group received corresponding interventions. After the intervention, they participated in the parallel source memory test (Version B) to evaluate the effects of rTMS intervention.

### Source Memory Test

#### Materials

Formal research materials include 480 black-background color pictures of daily life objects that were used in the present study (Li et al., 2004). The specification of the pictures is 3.8 cm × 3.8 cm. Out of 480 pictures, 240 were used as stimulation materials of Version A, and the rest were used as the stimulation materials of Version B. Versions A and B served as parallel testing and were respectively used before and after the intervention. The possibility of Version A and Version B to be used as pretest material or posttest material was balanced among subjects.

Taking the programming of Version A as an example, 240 pictures were divided into six groups according to two matched properties in each picture – one is the size of the object (bigger or smaller than a shoebox in daily life) and another is animacy of the object (whether animate or not). Among the six groups, four groups served as old stimulation to present in the study phase, of which two out of four groups are used as the estimation of size and another two groups were used as the estimation of animacy. Pictures from the remaining two groups served as new pictures to present in the test phase.

This study had two degrees of difficulty: easy condition and hard condition. Under the easy condition, the elder subjects were required to learn all the pictures three times. Under the hard condition, all the pictures in the study phase were only presented once.

The pictures in the test phase included old pictures that had been learned before as well as new pictures. The entire testing process was divided into four blocks. For each block, two pictures were set as the embedding material at the beginning. Before the start of the formal experiment, each subject participated in a practice task. The practice materials were 16 other pictures.

#### Procedure

**FIGURE 2:**
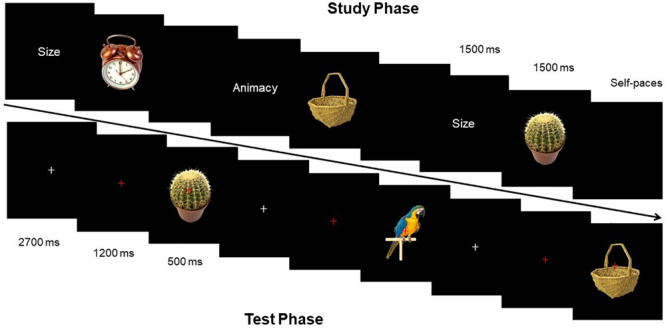
Schematic illustration of the stimuli and experimental design.

Source-Memory Test program used E-Prime compilation. The distance between subjects and the screen was 1 m, and the angle of stimulation was 2.3° × 2.6°. The flowcharts of the study phase and test phase were displayed as shown in Figure 2.

#### Study Phase

Before the presentation of each picture, clue words (size and animacy) were presented at first for 1500 ms. Then during the process of presenting stimulation pictures, subjects were required to learn pictures and estimate the size of objects (if the size of the object in the picture can be put in a shoebox or not) or estimate the animacy of objects (if the presented object in the picture has life or not). Each stimulation picture lasted for 1500 ms, and experimenters recorded the oral responses of all participants. After answering one picture, subjects pressed the spacebar to present the next clue word.

#### Test Phase

Fixation point “+” was always located at the center of the screen, with the “+” from white to red after 2,700 ms, to remind participants that a picture would appear soon. Red “+” lasted for 1,200 ms, and then the stimulation picture would show up for 500 ms. Subjects needed to determine the categories of each picture by keypress: (1) new picture; (2) picture in the study phase with size estimation; and (3) picture in the study phase with animacy estimation. Subjects made three responses by pressing three keys, including “size,” “animacy,” and “new.” Subjects were supposed to correctly estimate as soon as possible. Response keys were balanced among subjects. Before the start of the experiment, experimenters labeled the three keys on the keyboard. EEG was recorded during the test phase.

### Transcranial Magnetic Stimulation Protocol

According to the guidance of the rTMS International Safety Symposium, this study used 10 Hz rTMS to apply to subjects’ right DLPFC. The rTMS in the present study occurred at only one site, which was the right DLPFC. The reason we chose this site was that the right frontal cortex is especially associated with episodic retrieval, and we aimed to explore how rTMS could affect the retrieval phase of source memory. The intervention cycle was five times per week for a total number of 2 weeks. The intervention of each time lasted for 20 min. The stimulated brain region is the DLPFC of the right hemisphere. Based on EEG International 10-20 System, this study focused on the F4 locus. For the rTMS group, we set the coil and scalp into tangent placement, and the direction of the coil handle was paralleled to the median sagittal plane. For the sham group, we placed the coil perpendicular to the scalp.

We used MagPro X100 MagVenture Magnetic Stimulator and an 8-shaped quenchable coil (MFC-B65). Before the start of the intervention, we measured the motion valve limit for each participant. Motion valve limit was defined as the minimum stimulus output value that has a 50% chance to cause the short-muscle contraction of contralateral thumb abduction, which means observing five times of contraction of short-muscle thumb abduction in consecutive 10 times of stimulation – this output strength value was the motion valve limit for subjects.

The motion valve limit was measured by electromyography (EMG), and the contraction valve limit was 50 μV. For the rTMS group, the output value for each participant was set as 90% of the motion valve limit. The rTMS frequency was set as 10 Hz, with a stimulus cycle of 5-s pulse and 25-s interval each time, for a total number of repetitive 40 times. Each subject received 2,000-rTMS pulses per time of intervention for 20 min. For the sham group, the setting of parameters in the sham group was the same as in the rTMS group, but the coil and scalp were in vertical placement (Kim et al., 2012).

### Electroencephalogram Recording

The extended International 10-20 System elastic cap with 62 Ag/AgCl scalp electrodes and NeuroScan system were used to record the EEG data. Impedance of all electrodes was kept below 5 kΩ. The vertical electrooculograms (EOGs) was monitored by a couple of electrodes below and above the right eye, and the horizontal EOGs were monitored by a pair of electrodes at the external canthi of the two eyes. Neuroscan Scan 4.5 software was used for data analysis. The EEG data (sampling rate 500 Hz, ranging 0.05–100 Hz) was re-referenced to the average of the both right and left side mastoid electrode and filtered off-line with a bandpass of 0.05–40 Hz. EOG blink artifacts were redressed with a linear regression estimate (Semlitsch et al., 1986). Epochs were created from –200 to 1600 ms relative to stimulus onset, and 200 ms before the stimulus was the baseline period. Trials holding voltages over ± 100 μV were excluded from the analysis before averaged. The numbers of average superposition for the EEG total average waveform were all greater than 17.

### Data Analysis

#### Behavioral Data Analysis

All data analyses were performed by SPSS 19.0 (IBM Corporation, Somers, NY, United States), and all dependent variables for analysis were tested and met the criteria for a normal distribution. The gender factor was examined using the chi-square test. Independent-samples *t*-test was conducted to examine the group differences in demographic and clinical characteristics on the baseline. For memory performance, the source discrimination (Pr) of the source memory test was calculated. For the discrimination score for source memory, we defined Size as the target source (Hit: Size items correct judged as “Size”) and the Animacy as the lure source (False alarm: Animacy items false judged as “Size”) (Mollison and Curran, 2012; Zheng et al., 2016). Source discrimination was calculated by subtracting the false alarm rate from the hit rate. The results would remain the same if the target source and lure source were switched. To analyze the intervention effect of rTMS, repeated-measures analyses of variance (rmANOVAs) were conducted to calculate the source Pr and reaction time (RT) separately under easy condition,^1^ of which the between-subject factor is Group (rTMS vs. sham) and the within-subject factor is Time (pre vs. post). Greenhouse–Geisser was used to make a correction if the data was non-sphericity. The corrected *p*-value, uncorrected degree of freedom (df), and effect sizes (partial eta square: η^2^*_*p*_*) would be reported. If the rmANOVAs showed a significant interaction of Group × Time, *t*-test would be used to conduct the comparison within each group. The alpha level was set to 0.05 for all analyses.

#### Event-Related Potential Data Analysis

Although not all of the raw ERP data met the criteria for a normal distribution, the ANOVA has been proved as a robust method to non-normality distribution data (Schmider et al., 2010; Ferreira et al., 2012; Mena et al., 2017). The selection of electrodes and latency intervals was chosen based on previous related studies and visual inspection of the present ERP waveforms (Li et al., 2004; Zheng et al., 2015a, b). Six clusters of electrode sites along the anterior–posterior axis were chosen, and each cluster averaged the amplitudes of the three adjacent electrode sites: left frontal (F1, F3, and F5), left central (C1, C3, and C5), left parietal (P1, P3, and P5), right frontal (F2, F4, and F6), right central (C2, C4, and C6), and right parietal (P2, P4, and P6). The old/new effect latency intervals of 300–500, 500–700, and 700–1500 ms were selected for the observation of the right frontal old/new effect, the left parietal old/new effect, and the late negativity effect, respectively. The old/new effect was quantified by calculating the average amplitudes over each time window.

All ERP data were analyzed in two stages. The first-stage analyses were aimed to determine the presence of a classical old/new effect and the change of an old/new effect through the intervention within each group. In the first stage, ERP data were analyzed in the rTMS group and the sham group, separately. Please note that the ERP old/new effect by comparing the ERPs of source corrected items with the ERPs of correct rejection items. The rmANOVAs in the first stage involved two within-subject factors: Time (pre vs. post) and Condition (source correct vs. correct rejection). For the 300–500 ms, rmANOVAs were performed on the right frontal electrode sites; for the 500–700 ms, rmANOVAs were performed on the left parietal electrode sites. If the rmANOVAs showed a significant interaction of Time × Condition, we would decompose the Time factor and conduct the *t*-test to quantify the old/new effect in both pre-intervention and post-intervention. For the 700–1500 ms, considered the late reversed old/new effect, which showed a widespread topographic distribution, we conducted rmANOVAs with four within-subject factors of Time (pre vs. post), Condition (source correct vs. correct rejection), Location (frontal vs. central vs. parietal), and Hemisphere (left vs. right). If the initial rmANOVAs showed a significant interaction involving Time and Condition, we would conduct subsidiary ANOVAs separately on the pre-intervention time point and post-intervention. For the subsidiary analysis that showed interactions involving the factor of Condition, we would conduct a simple effect analysis to further determine the quantity of an old/new effect at the different locations. Topographic maps of the old/new effect were formed by subtracting the ERPs of the correct rejection trails from the ERPs of the source correct trails.

Second-stage analysis aimed to determine whether there was an rTMS-specific intervention effect, by comparing the changes of an old/new effect from pre-intervention to post-intervention between two groups. Therefore, instead of raw amputation of different conditions, the different waveforms were conducted as the dependent variable. The different waveforms were the raw amplitude of source correct items minus the raw amplitude of correct rejection items, which was the magnitude of an old/new effect. A repeated-measures ANOVAs with the within-subject factor of Time (pre vs. post) and the between-subject factor Group (rTMS vs. sham) were performed to determine the intervention effect on the right frontal old/new effect and the left parietal old/new effect. If the rmANOVAs showed a significant interaction with Group × Time, we would conduct further comparisons separate in two groups. For the 700–1500 ms, rmANOVAs with the within-subject factors of Time (pre vs. post), Location (frontal vs. central vs. parietal), Hemisphere (left vs. right), and the between-subjects factor Group (rTMS vs. sham) were performed to detect the intervention effect on the late reversed old/new effect.

## Results

### Demographic and Clinical Characteristics

Participants from both groups show no statistically significant differences in age, gender, years of education, and the score of MoCA-BJ (all *p* > 0.05). See Table 1 for demographic details.

**TABLE 1:** Demographic characteristics and the score of MoCA-BJ at baseline for rTMS and sham group (means ± SD).

	rTMS group (*n* = 16)	Sham group (*n* = 16)	*p*-value
Age (years)	67.88 ± 5.51	66.50 ± 4.27	0.437
Gender (female/male)	14/2	13/3	0.585
Education (years)	12.56 ± 3.01	11.88 ± 2.99	0.522
MoCA-BJ	28.31 ± 1.35	28.75 ± 1.48	0.390

*rTMS, repetitive transcranial magnetic stimulation; MoCA-BJ, Beijing version of the Montreal Cognitive Assessment.*

### Behavioral Results

#### Source Discrimination

**FIGURE 3:**
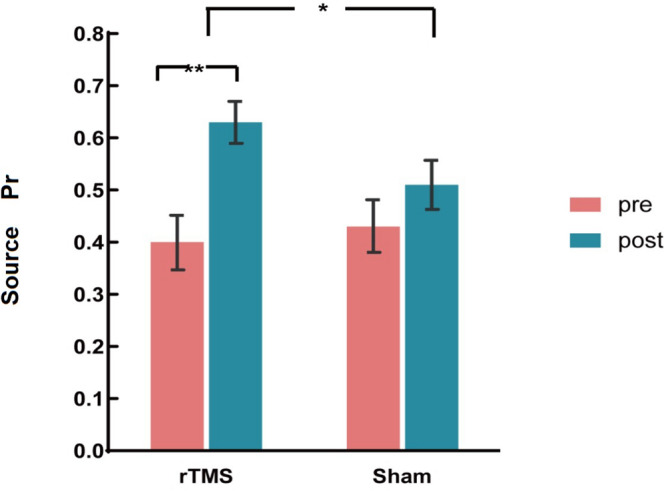
The plots display the source discrimination in each group [repetitive transcranial magnetic stimulation (rTMS) group and sham group)] at pre-intervention and post-intervention. Error bars represent standard errors of mean. *p < 0.05; **p < 0.01.

The ANOVAs showed a significant main effect of Time [*F*(1, 30) = 45.18, *p* < 0.001, η^2^*_*p*_* = 0.601] and a significant two-way interaction of Group × Time [*F*(1, 30) = 14.47, *p* = 0.001, η^2^*_*p*_* = 0.325]. A further interaction analysis using *t*-test observed that post-source memory Pr of the rTMS group was significantly higher than the pre-Pr [*t*(15) = 8.34, *p* < 0.001)], whereas the post-source memory Pr of the sham group has no significant difference with the pre-Pr [*t*(15) = 1.68, *p* = 0.096] (see Figure 3). Consistent with our hypothesis, we conclude that there was a gain of rTMS intervention in the source memory test for the rTMS group compared with the sham group.

#### Reaction Time

The ANOVAs revealed only a marginal significant main effect of Time [*F*(1, 30) = 3.72, *p* = 0.064, η^2^*_*p*_* = 0.114], which suggested that rTMS intervention did not affect the response time.

### Event-Related Potential Results

#### Repetitive Transcranial Magnetic Stimulation Group

##### 300–500 ms

**FIGURE 4:**
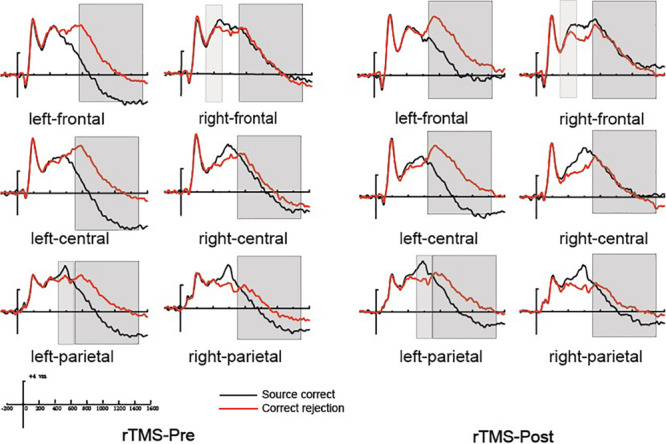
The grand average event-related potential (ERP) waveforms for source correct items (black) and correct rejection items (red) for pre-intervention and post-intervention in the repetitive transcranial magnetic stimulation (rTMS) group at six clusters of electrode sites, from –200 to 1600 ms. The scale bars indicate the time windows used for the statistical analyses (300–500, 500–700, and 700–1500 ms). The positive voltages are plotted upwards.

Previous studies demonstrated that the 300–500 ms old/new effect was most pronounced at the right frontal scalp region in older adults; therefore, we conducted the analysis focus on the right frontal location. At the right frontal location, the initial repeated-measures ANOVAs with the within-subject factors of Time (pre vs. post) and Condition (source correct vs. correct rejection) uncovered a marginal main effect of Condition [*F*(1, 15) = 4.17, *p* = 0.059, η^2^*_*p*_* = 0.217], demonstrating a faintish right frontal old/new effect (see Figure 4). No interaction effect involving Time suggested that the difference between source correct items and correct rejection items has no difference at pre-intervention and post-intervention in the rTMS group. Figures 6A,B delineated the topographic maps of the old/new effect.

##### 500–700 ms

The 500–700 ms old/new effect was most pronounced at the left parietal scalp region in older adults; therefore, we conducted the analysis focus on left parietal location. The initial ANOVA revealed a significant main effect of Condition [*F*(1, 15) = 17.86, *p* = 0.001, η^2^*_*p*_* = 0.543] and a significant Time × Condition interaction [*F*(1, 15) = 11.35, *p* = 0.004, η^2^*_*p*_* = 0.431]. As revealed by *t*-test, the ERPs of source correct items were significantly more positive than those of correct rejection items for the pre-intervention test [*t*(15) = 3.38, *p* = 0.004], and the differences between the ERPs of source correct items and those of correct rejection items were also significant for the post-intervention test [*t*(15) = 5.39, *p* < 0.001] (see Figure 4). The outcomes indicated that the left parietal old/new effect was robust both before and after the rTMS intervention. Moreover, the left parietal old/new effect was larger for post-intervention than that for pre-intervention [*t*(15) = 3.37, *p* = 0.004]. Figures 6A,B delineated the topographic maps of the old/new effect.

##### 700–1500 ms

The initial ANOVAs demonstrated a two-way interaction of Time × Condition [*F*(1, 15) = 5.04, *p* = 0.042, η^2^*_*p*_* = 0.265]. Subsidiary analysis revealed that correct rejection items were significantly more positive going than source correct items at the left frontal (*p* < 0.001), left central (*p* < 0.001), left parietal (*p* < 0.001), and right parietal (*p* = 0.042) scalp regions at pre-intervention stage. For the post-intervention, subsidiary analysis reported significant reversed old/new effects at the left frontal (*p* < 0.001), left central (*p* = 0.003), and left parietal (*p* = 0.015) scalp regions. More importantly, these reversed old/new effects were significantly attenuated after rTMS intervention as manifested in the Time × Condition interaction (see Figure 4). Figures 6A,B delineated the topographic maps of the old/new effect.

#### Sham Group

##### 300–500 ms

**FIGURE 5:**
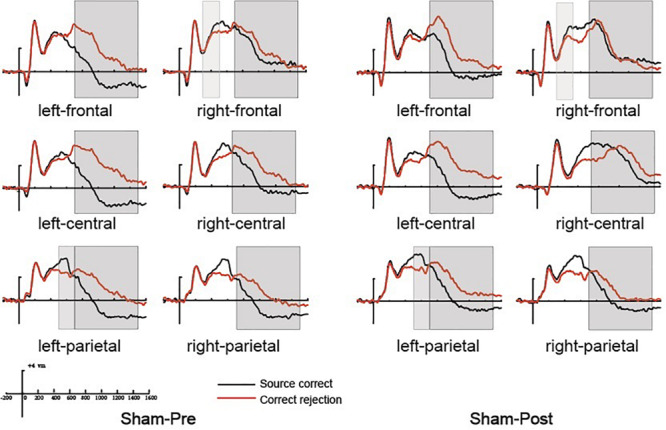
The grand average event-related potential (ERP) waveforms for source correct items (black) and correct rejection items (red) for pre-intervention and post-intervention in the sham group at six clusters of electrode sites, from –200 to 1600 ms. The scale bars indicate the time windows used for the statistical analyses (300–500, 500–700, and 700–1500 ms). The positive voltages are plotted upwards.

**FIGURE 6:**
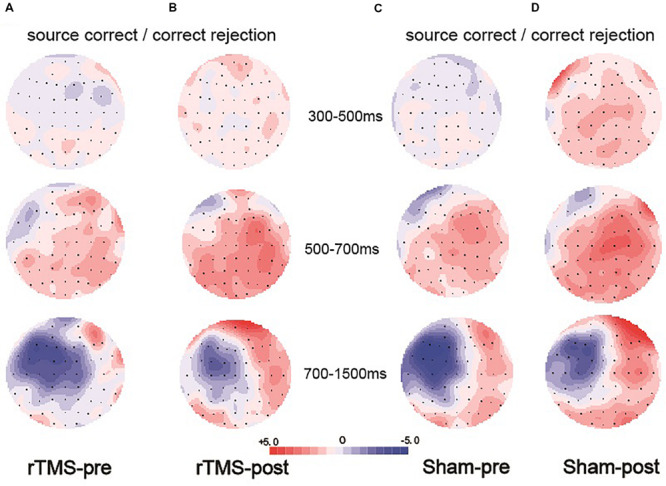
Topographic maps of the old/new effect in three time windows (300–500, 500–700, and 700–1500 ms), which were formed by subtracting the event-related potentials (ERPs) of the correct rejection items from the ERPs of the source correct items. **(A)** Topographic maps for the pre-intervention test in the repetitive transcranial magnetic stimulation (rTMS) group. **(B)** Topographic maps for the post-intervention test in the rTMS group. **(C)** Topographic maps for the pre-intervention test in the sham group. **(D)** Topographic maps for the post-intervention test in the sham group; the scale bar shows the amplitude range.

At the right frontal location, the initial repeated-measures ANOVAs with the within-subject factors of Time (pre vs. post) and Condition (source correct vs. correct rejection) revealed a marginal main effect of Condition [*F*(1, 15) = 7.51, *p* = 0.015, η^2^*_*p*_* = 0.334], suggesting a reliable right frontal old/new effect (see Figure 5). No interaction effect involving Time suggested that the difference between source correct items and correct rejection items has no difference at pre-intervention and post-intervention in the sham group. The topographic maps of the old/new effect are illustrated in Figures 6C,D.

##### 500–700 ms

The initial ANOVA revealed a significant main effect of Condition [*F*(1, 15) = 16.85, *p* = 0.001, η^2^*_*p*_* = 0.529] and a significant main effect of Time [*F*(1, 15) = 6.22, *p* = 0.025, η^2^*_*p*_* = 0.293]. The main effect of Condition indicated source correct items and correct rejection items revealed a robust left parietal old/new effect at pre-intervention and post-intervention (see Figure 5). No significant interaction involving Time indicated the left parietal old/new effect was not different between pre-intervention and post-intervention. The topographic maps of the old/new effect are illustrated in Figures 6C,D.

##### 700–1500 ms

A comparison of source correct and correct rejection items indicated a significant reversed old/new effect at pre-intervention and post-intervention (see Figure 5). The initial ANOVA revealed a three-way interaction of Condition × Location × Hemisphere [*F*(2, 30) = 9.18, *p* = 0.001, η^2^*_*p*_* = 0.380], subsidiary analysis showed that correct rejection items were more positive than source correct items significantly at the left frontal (*p* < 0.001), left central (*p* < 0.001), right central (*p* = 0.008), left parietal (*p* < 0.001), and right parietal (*p* < 0.001) scalp regions. Meanwhile, no interaction effect involving Time suggested that the difference between source correct items and correct rejection items has no difference at pre-intervention and post-intervention. The topographic maps of the old/new effect are illustrated in Figures 6C,D.

#### Between-Groups Comparison

##### 300–500 ms

**FIGURE 7:**
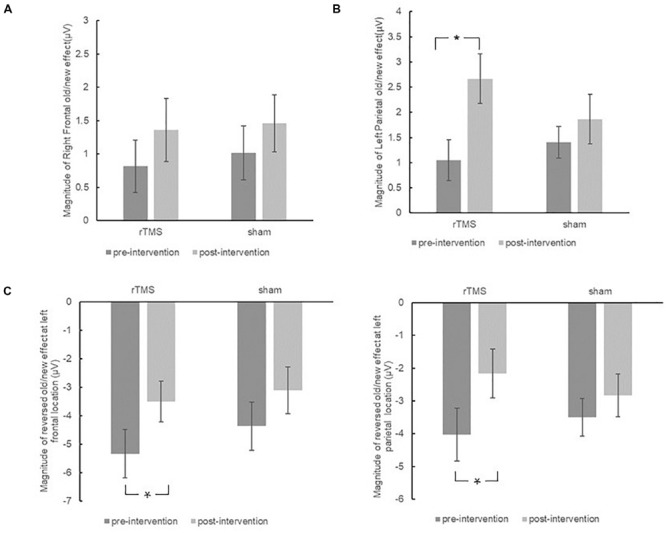
The plots display the magnitude of right frontal old/new effect **(A)**, left parietal old/new effect **(B)**, and reversed old/new effect at left frontal sites and left parietal sites **(C)** for pre-intervention and post-intervention in the repetitive transcranial magnetic stimulation (rTMS) group and sham group. Error bars represent standard errors of mean. *p < 0.05.

The initial ANOVA revealed neither significant main effect nor interaction effect, further validating the findings from the separate group analysis that the right frontal old/new effect did not change throughout the intervention in both the repetitive transcranial magnetic stimulation (rTMS) and sham groups (see Figure 7A).

##### 500–700 ms

The different pre-changing versus post-changing patterns of left parietal old/new effects revealed in the above each group analysis were confirmed by a marginal significant Time × Group interaction [*F*(1, 30) = 3.26, *p* = 0.081, η^2^*_*p*_* = 0.098]. As revealed by *t*-test, the left parietal old/new effect was significantly increased after rTMS intervention [*t*(15) = 3.37, *p* = 0.004]. Whereas in the sham group, the left parietal old/new effect has no significant difference between pre-intervention and post-intervention [*t*(15) = 1.09, *p* = 0.294]. The results suggested that rTMS intervention modulated the left parietal old/new effect; however, there was no such beneficial effect in the sham group (see Figure 7B).

##### 700–1500 ms

The initial ANOVA only revealed a marginal significant Time × Location × Hemisphere interaction [*F*(2, 58) = 2.42, *p* = 0.098, η^2^*_*p*_* = 0.077]. Subsidiary analysis revealed that the negative old/new effect was decreased after intervention at the left frontal (*p* = 0.008), right central (*p* = 0.024), and left parietal (*p* = 0.037) scalp regions. There was no interaction effect involving Time × Group, suggesting the attenuation of these reversed old/new effects was the same in the rTMS group and the sham group (see Figure 7C).

#### Summary of Event-Related Potential Findings

In sum, both the rTMS group and the sham group showed a faintish 300–500 ms right frontal old/new effect before the intervention, whereas after the intervention, the early right frontal old/new effect remained stable. For the 500–700 ms time window, both groups showed a robust left parietal old/new effect before and after the intervention; however, the changes of a left parietal old/new effect between pre-intervention and post-intervention showed different patterns in the rTMS and sham groups. For the rTMS group, the left parietal old/new effect became significantly larger after intervention, but it remained unchanged in the sham group. For the 700–1500 ms, a significant reversed old/new effect presented in both the rTMS and sham groups at pre-intervention. In addition, a similar attenuation after intervention happened in both groups. Taken all together, the rTMS intervention modulated the left parietal old/new effect but not did affect the early right frontal old/new effect and later reversed old/new effect.

## Discussion

In the present study, we investigated whether the application of rTMS could enhance the source memory performance in healthy older adults in a randomized sham-controlled intervention study. Besides, we used ERPs to explore the specific process modulated by rTMS during the source memory retrieval. Participants were randomly assigned to the rTMS group or the sham group. The rTMS group received 10 sessions of 10 Hz rTMS applied on the right DLPFC, and the sham group received 10 sessions of sham stimulation. Both groups performed a source memory test before and after the intervention.

For the behavioral results, the present study indicated that rTMS significantly improved source memory discrimination. Episodic memory, especially the source memory, is the most vulnerable memory ability in the course of aging. The present results supported the rTMS as a promising non-invasive intervention tool to improve the delicate ability even in healthy adults. The improvement of source memory was consistent with previous studies that implemented the multi-session off-line rTMS in MCI and Alzheimer’s disease patients (Cotelli et al., 2012; Drumond Marra et al., 2015; Koch et al., 2018). Only few studies investigated the rTMS off-line effect in healthy adults, which have not found that rTMS significantly improves episodic memory in healthy older adults (e.g., Vidal-Piñeiro et al., 2014; Davis et al., 2017), perhaps because those studies have used a single-session stimulus protocol. The present findings suggested multi-session off-line stimulus protocol could produce accumulated intervention effects in healthy older adults. However, unfortunately, the present study did not track the memory changes during the 10 sessions of interventions. To directly examine this speculation, future research may consider setting up consecutive tests to investigate when the intervention effects may appear and how the effects would develop over time. These are very important for future rTMS studies to find the right parameters in order to bring beneficial effects for older adults’ memory functions.

In addition, the present results showed that 10 Hz rTMS stimulus is an effective parameter to enhance episodic memory. A meta-analysis indicated that off-line 20, 10, and 5 Hz rTMS were all effective to enhance episodic memory (Yeh and Rose, 2019). The present study further supported the high-frequency rTMS could promote source memory. Future studies could pay more attention to not only the efficacy but also the safety of various high-frequency rTMS in the elderly. As we mentioned in the *Introduction*, DLPFC is the main target locus in most TMS protocols. Besides, as indicated by the hemispheric encoding/retrieval asymmetry (HERA) model (Tulving et al., 1994), the left frontal cortex is specially engaged with episodic encoding, whereas the right frontal cortex is especially associated with episodic retrieval. For our study, we aimed to explore the retrieval phase of source memory; therefore, we chose the right DLPFC as the targeting location. For the healthy older adults, the present results indicated that the right DLPFC was an effective targeted location to improve episodic memory.

As for the ERP results, we found that the right frontal old/new effect, which was considered as the neural indicator of familiarity, remained unchanged throughout the intervention in both the rTMS group and the sham group. This result was within expectation, because the familiarity-based retrieval process remained relatively intact in the elderly (Howard et al., 2006; Zheng et al., 2016), and it hardly played a decisive role in the source memory task, so the improvement in the performance of the source memory in the present study was not related to the familiarity-based ERP component. The left parietal old/new effect was improved in the rTMS group, but not in the sham group. The magnitude of the left parietal old/new effect has been appeared to adjust with the retrieval of associated contextual information (Donaldson and Rugg, 1998), and it is proportional to the amount of information have been retrieved (Vilberg et al., 2006; Vilberg and Rugg, 2009). Previous studies have suggested that the decline in episodic memory ability was due to impaired recollection with aging (Mark and Rugg, 1998; Prull et al., 2006; Bugaiska et al., 2007). High-performing older adults have been found to exhibit intact recollection relative to the older adults with low performance, which is to say, successful aging was inseparable from the retention of recollection process during retrieval (Duarte et al., 2006; Dockree et al., 2015). Along this line, we speculated that the participants after rTMS could have recollected more critical details that supported their better source memory decisions.

For the late reversed old/new effect, the present results demonstrated a similar attenuated pattern in the rTMS group as in the sham group. Consistent with some previous studies (Wegesin et al., 2002; Dulas and Duarte, 2013; James et al., 2016), the present ERP results showed co-existence of the left parietal old/new effect and late reversed old/new effect. In some studies, the late reversed started earlier, and even overlapped with the left parietal old/new effect, resulting in the disappearance of the left parietal old/new effect (Horne et al., 2020). A number of previous studies have suggested that the reversed old/new effect in older adults reflected the process of reconstruction or continued evaluation of retrieval information in the source-memory task, as it does in young adults (Wiese et al., 2012). However, compared with young adults, the late reversed old/new effect had a remarkably different topography distribution in older adults. Instead of as being typically posteriorly distributed in the young, the reversed old/new effect in older adults was more widespread, central, and left lateralized distributed. The differential scalp distribution suggested that the reversed old/new effect in older adults was unlikely the same component in young adults (Mecklinger et al., 2016). Li et al. (2004) suggested that this reversed old/new effect in older adults may reflect the search processes aiming at the recovery of perceptually visual information, a relatively low-level retrieval strategy to compensate for the impoverished conceptual retrieval process. Therefore, this negativity component may be specific to the elderly as a neural indicator associated with recollection (Dulas and Duarte, 2013). Given that the left parietal old/new effect supported recollection-based processing in both young and old adults, whereas the later reversed old/new effect could only specifically correlate to older adults’ recollection retrieval, the present results supported the idea that rTMS selectively enhances more youth-like recollection processes, rather than the aging-specific retrieval processing, making older people more similar to younger people in the process of episodic memory retrieval.

Several limitations should be noted. First, the localization of the stimulated brain region (right DLPFC) depended on the EEG International 10-20 System, which was F4. However, previous studies showed rTMS yielded smaller effect sizes when targeting location with the 10–20 EEG positioning system than which are obtained with individual fMRI-guided TMS neuronavigation (Sack et al., 2009). Second, we only used the sham stimulus as a control condition but did not set a control stimulation site. Future studies should assign an active control group with rTMS over other scalp site to further identify the specific connection between right DLPFC and episodic retrieval. Third, in the statistical analysis of present ERP data, the between-group interaction for the left parietal old/new effect was only marginally significant, and we did not find a significant correlation between the change in the left parietal old/new effect and the change in the source memory, which may be caused by a small sample size. Therefore, the current results should be interpreted with caution owing to the relatively small sample size. Future research should pay close attention to these issues.

Taken together, the present results suggested that rTMS could be an effective intervention to improve source memory performance in healthy older adults, and it selectively facilitated the youth-like recollection process during retrieval.

## Data Availability Statement

The datasets generated for this study are available on request to the corresponding author.

## Ethics Statement

The studies involving human participants were reviewed and approved by the Ethics Committee of the Institution of Psychology, Chinese Academy of Sciences. The patients/participants provided their written informed consent to participate in this study. Written informed consent was obtained from the individual(s) for the publication of any potentially identifiable images or data included in this article.

## Author Contributions

XC analyzed the data and drafted the manuscript. WR collected and analyzed the data. ZZ interpreted and revised the manuscript. JL conceived and designed the study and interpreted and revised the manuscript.

## Conflict of Interest

The authors declare that the research was conducted in the absence of any commercial or financial relationships that could be construed as a potential conflict of interest.

## Footnotes

1 The results of the hard condition were unreported owing to the floor effect of the accuracy.
